# Assessment of the effect of tricaine (MS-222)-induced anesthesia on brain-wide neuronal activity of zebrafish (*Danio rerio*) larvae

**DOI:** 10.3389/fnins.2024.1456322

**Published:** 2024-09-24

**Authors:** Nils Ohnesorge, Jenny Wilzopolski, Matthias Steinfath, Lars Lewejohann, Stefanie Banneke, Céline Heinl

**Affiliations:** ^1^German Centre for the Protection of Laboratory Animals (Bf3R), German Federal Institute for Risk Assessment (BfR), Berlin, Germany; ^2^Institute of Animal Welfare, Animal Behavior and Laboratory Animal Science, Freie Universität Berlin, Berlin, Germany

**Keywords:** zebrafish, brain, nociception, behavior, anesthesia, tricaine

## Abstract

Fast and effective anesthesia is the key for refining many invasive procedures in fish and gaining reliable data. For fish as for all vertebrates, it is also required by European law to reduce pain, suffering, and distress to the unavoidable minimum in husbandry and experiments. The most often used substance to induce anesthesia in zebrafish is tricaine (MS-222). When properly prepared and dosed, tricaine causes rapid loss of mobility, balance and reaction to touch. These signs are interpreted as a stage of deep anesthesia although its effects on the central nervous system have not convincingly been shown. Therefore, it might be possible that tricaine first acts only on the periphery, resulting in a paralyzed instead of an anesthetized fish. This has severe implications for animals undergoing procedures. To investigate the effects of tricaine on the central nervous system, we used zebrafish larvae [Tg(*elavl3*:H2B-GCaMP6s)] at 4 days post fertilization (dpf), expressing a calcium indicator (GCaMP6s) in all neurons, that allows monitoring and quantifying the neuronal activity. After treating larvae with 168 mg/L tricaine, a rapid loss of neuronal activity in the forebrain was observed in confocal microscopy. In contrast, only mild effects were seen in the midbrain and hindbrain. In conclusion, the different larval brain areas showed differences in the sensitivity to tricaine treatment. The effects on the central nervous system are indicative of tricaine’s anesthetic function and are consistent with behavioral observations of inactivity and unresponsiveness to touch.

## 1 Introduction

Only in recent years, fish welfare has come into focus as increasing evidence suggests that fish have perception capacities that resemble those of other vertebrates, i.e., mammals (Sneddon, 2015; Ohnesorge et al., 2021). Providing proper anesthesia is therefore an important requirement for many animal experiments to ensure animal welfare, wellbeing and data reliability. Research interest in the model of zebrafish (*Danio rerio*) markedly increased in the last 10 years, reflected in a rise of scientific publications per year from 2,724 to 4,423 (number of hits in PubMed search term “zebrafish” accessed on September 20, 2023). Thus, there is an urgent need to improve our knowledge about the mechanism of action of anesthetic drugs in fish. Today a multitude of anesthetic protocols exists for zebrafish (Martins et al., 2018; Jorge et al., 2021). There has been progress in identifying safe doses for varying agents (Martins et al., 2016). Whether these can provide deep anesthetic states with unconsciousness, loss of sensation, analgesia and muscle relaxation or only a paralytic state is difficult to determine by sole observation of behavior and needs further elucidation. For isoeugenol, it was recently shown in goldfish that it might only be suitable for light anesthesia but not for severe procedures (Machnik et al., 2023). This demonstrates that in order to avoid pain or distress for the animals, it is important to investigate commonly used anesthetics in zebrafish with other methods to determine the level of anesthesia, to gauge their effect on the central nervous system and how quickly these effects take place.

So far, characterization of anesthetics in zebrafish mainly relied on behavioral observations (Collymore, 2020). The states of anesthesia from light to deep are defined by loss of balance, cessation of movement, decrease in opercular movement, reduced heart rate, and loss of reflex reactivity (e.g., visual, tactile) (Summerfelt and Smith, 1990; Martins et al., 2016). Currently the main characteristics of anesthetics are defined as the time until these criteria are reached, recovery after wash out and the safety margin of the drugs used to avoid over- or underdosing (Collymore, 2020). However, these behavioral observations cannot safely rule out that muscle excitability ceases before the central nervous system is affected. This would leave the animal initially awake and aware during experiments, creating unnecessary pain and distress.

Tricaine (MS-222) is the most often applied anesthetic in zebrafish, usually in the form of immersion bath for general anesthesia (Lidster et al., 2017; Jorge et al., 2021). It is well characterized in terms of safe dose ranges, stability, and side effects (Martins et al., 2018; Collymore et al., 2014; Katz et al., 2020). Pharmacologically MS-222 is a local anesthetic that acts mostly by blocking voltage gated sodium channels thus reducing action potentials resulting in muscle relaxation (Frazier and Narahashi, 1975; Attili and Hughes, 2014). In *Xenopus laevis* tadpoles, it was shown that both motor and sensory nerve activity can be blocked (Ramlochansingh et al., 2014). Still, its anesthetic effect on the whole brain activity has not been systematically investigated in zebrafish.

A recent study has shown that Tricaine reduces the neuronal activity in the pretectum as well as in the hindbrain in zebrafish larvae after a visual stimulation (Leyden et al., 2022). In this study, a loss of functional connectivity between the pretectum and the hindbrain was observed. This loss is proposed to be a defining characteristic of anesthesia and possibly a mechanism behind the loss of consciousness in humans (Bonhomme et al., 2012; Nallasamy and Tsao, 2011; Yang et al., 2021; Hudetz, 2012). Still, the neuronal effect of tricaine in the central nervous system on other brain areas potentially involved in nociception is still missing. An identification of the brain regions involved and a detailed quantification of neuronal dynamics across the zebrafish brain are necessary to better understand anesthesia in these animals (Loring et al., 2020). As there is no single switch for (un)consciousness, investigations are needed for tricaine and other anesthetic agents to elucidate their mode of action in zebrafish or fish in general (Bonhomme et al., 2012). Furthermore, other pharmacodynamic effects of, e.g., tricaine, essential for anesthesia, on the central nervous system remain elusive. A recent study showed that 14 out of 15 zebrafish examined lost their nociceptive withdrawal reflex under tricaine anesthesia (Jorge et al., 2021). A similar effect was observed for koi (*Cyprinus carpio*). With increasing concentrations of tricaine the response to noxious stimulation decreased in these animals (Stockman et al., 2013). This indicates that tricaine might also have an analgesic effect during anesthesia via unknown neuronal mechanisms, making it a good candidate for use during painful surgical procedures in these species.

In this study, we tested the effect of tricaine on the spontaneous brain activity of zebrafish larvae at 4 days post fertilization (dpf). Larvae at 4 dpf are a suitable system, as they are still small enough and transparent to allow for whole brain imaging and are sufficiently developed to study neural circuits of complex behaviors. We assumed that in contrast to paralysis anesthetic states are characterized by a reduced spontaneous activity in the whole brain, allowing to assess the depth of anesthesia in comparison to muscle activity and behavioral indicators. To investigate this, we quantified neuronal calcium concentrations via a transgenic, panneuronal reporter. There the application of tricaine should result in lasting reduction of activity, but the time until this effect takes place needed to be elucidated.

## 2 Materials and methods

### 2.1 Animals

Wildtype Tübingen (TU) obtained from the European Zebrafish Resource Centre (EZRC) and a transgenic line expressing a fluorescent, calcium reporter in all neurons Tg(*elavl3*:H2B-GCaMP6s)^jf5^ in a TU genetic background with *mitfa*^−/−^ to avoid pigmentation were used (Freeman et al., 2014). The fish were raised under standard conditions (28°C, pH 7.5, conductivity 500 μS/cm, 14/10 h light/dark cycle, holding density up to 5 fish per L) with temperature, pH and conductivity being continuously monitored (Goodwin et al., 2016; Alestrom et al., 2020). Nitrogen compounds were measured weekly to ensure low levels of NH_3_/NH_4_^+^ < 0.1 mg/L, NO_2_^−^ < 0.3 mg/L, and NO_3_^−^ < 25 mg/L (Alestrom et al., 2020). Adult fish were fed twice daily with dry food (Sparos) and as enrichment once per day with artemia (Sanders). Every 3 years, TU fish lines were replaced with new ones obtained from the EZRC to avoid creation of sublines.

For experiments, zebrafish eggs were collected in 10 cm petri dishes with E3 embryo medium containing 5.03 mM NaCl, 0.17 mM KCl, 0.33 mM CaCl_2_, and 0.33 mM MgCl_2_ with a maximum of 50 eggs per dish. Embryos were staged, and normal development was confirmed under an Olympus stereo microscope (SZX16) at 4, 24, and 96 h post fertilization (hpf) (Kimmel et al., 1995). Experiments were conducted at 4 and 5 dpf. Experiments were exempt from approval by local German authorities (LAGeSo) but the zebrafish husbandry was authorized (ZH 181). Zebrafish were kept in accord with EU directive 2010/63/EU on the protection of animals used for scientific purposes.

A total of 264 larvae was used for the experiments. For the behavioral analysis, 200 wild-type larvae were used, each with 100 larvae aged 4 or 5 dpf.

For quantification of brain activity 33 transgenic larvae were used in the final assessment, with 20 in the control and 13 in the treatment group. In addition, several larvae were excluded from the final assessment due to poor image quality (5), strong movements of the larvae during imaging (2), lack of blood flow after imaging (1), and use of alternative tricaine treatments (8, Supplementary Figure 1). Furthermore, 15 larvae were used in pilot experiments to establish mounting and imaging settings.

### 2.2 Tricaine preparation

Two different batches of pharmaceutical grade (MS-222, PharmaQ) and one batch of chemical grade (Sigma-Aldrich, #E10521) tricaine methanesulphonate were acquired as powder and stored at room temperature. Tricaine stock solutions of 4 mg/mL were prepared and Tris buffered to pH 7. Aliquots were frozen and stored at −20°C. For experiments, daily new stocks were freshly thawed and diluted with E3 medium to the required concentration for immediate use.

All experiments were performed with the same batch of MS-222 (PharmaQ), except for five larvae that were imaged while treated with the second batch of PharmaQ and three larvae that were imaged with chemical grade tricaine of Sigma-Aldrich (Supplementary Figure 1).

### 2.3 Observation of behavior

To assess the behavioral effectiveness of tricaine anesthesia, 4 or 5 dpf wildtype TU larvae were randomly picked and transferred to 400 μL E3 medium in a 12 well porcelain spot plate with one larva per well. After 5 min of habituation, 100 μL tricaine diluted in E3 were added to a final concentration of 168 mg/L. Larvae were observed for 60 s and the time point rounded up to nearest the 10 s of their last spontaneous activity, e.g., movements, tail flicks, or twitches, was assessed. Subsequently, larvae were gently touched with a metal poker to elicit a reflexive response (Leyden et al., 2022). This was repeated every 30 s until no reflexive response was observed.

All behavioral experiments were performed at room temperature of 24–25°C and a light intensity of 700 lux.

### 2.4 Neuronal imaging using confocal microscopy

Neuronal activity is associated with a strong influx of extracellular calcium, which was monitored in the brain of 4 dpf Tg(*elavl3*:H2B-GCaMP6s) larvae by confocal fluorescence imaging (Chen et al., 2013). An overview of the time course of the experiment is shown in Table 1.

**Table 1:** Overview of time course of neuronal imaging.

Timepoint (min)	Event
−30	Mounting larva
	Check vital signs of larva via brightfield microscopy
−10	Start confocal imaging, habituation
−5	Start determining baseline for 5 min
0	Adding treatment
20	Stop confocal imaging
	Check vital signs of larva in brightfield microscopy

Larvae were randomly picked, transferred to 1.5% low melting agarose (Roth #3810.3), placed in a drop of agarose on a 6 cm petri dish, with a dorsal orientation and overlaid with 9 mL E3 medium (Sabaliauskas et al., 2006). They were allowed to rest for 20 min at room temperature. Then, the dish was placed into the incubation chamber of the microscope, which was heated to 25–28°C.

Stacks of whole larval brains were taken in 6.9 μm steps of 35–40 optical slices every minute by time-lapse imaging with an upright Zeiss LSM 880 NLO using a 20x dipping objective (W Plan-Apochromat 20x/1.0 DIC VIS-IR). GCaMP6s was excited at 488 nm, and fluorescence emission was detected between 493 and 598 nm. Images (512 × 512 pixels) were acquired with a pixel dwell time of 1.5 μs and pixel size of 1.4 μm^2^. The confocal pinhole was set to one airy unit (31.9 μm). One frame was acquired within 0.5 s. Laser power applied to samples peaked at 28 μW, as measured via microscope slide power sensor (#S170C, Thorlabs) connected to an optical power and energy meter console (#PM100D, Thorlabs).

Since laser light startled larvae when confocal imaging commenced, the first 5 min of the measurements were considered as habituation period and thus were not taken into account. Another 5 min were recorded to determine the baseline neuronal activity of each larva. Treatment in form of either 1 mL E3 medium control or of pre-diluted tricaine (final concentration of 168 mg/L) was then added dropwise and equally distributed over the whole dish. Time-lapse imaging continued for another 20 min.

Vital signs in form of heartbeat and blood flow were monitored via brightfield microscopy for abnormalities before and after confocal imaging and only inconspicuous larvae were considered for further analysis. Control-treated larvae showed continuous eye movements throughout the experiment. When freed from agarose-embedding, control-treated larvae showed physiological swimming behavior after imaging.

### 2.5 Quantification of neuronal activity

**Figure 1:**
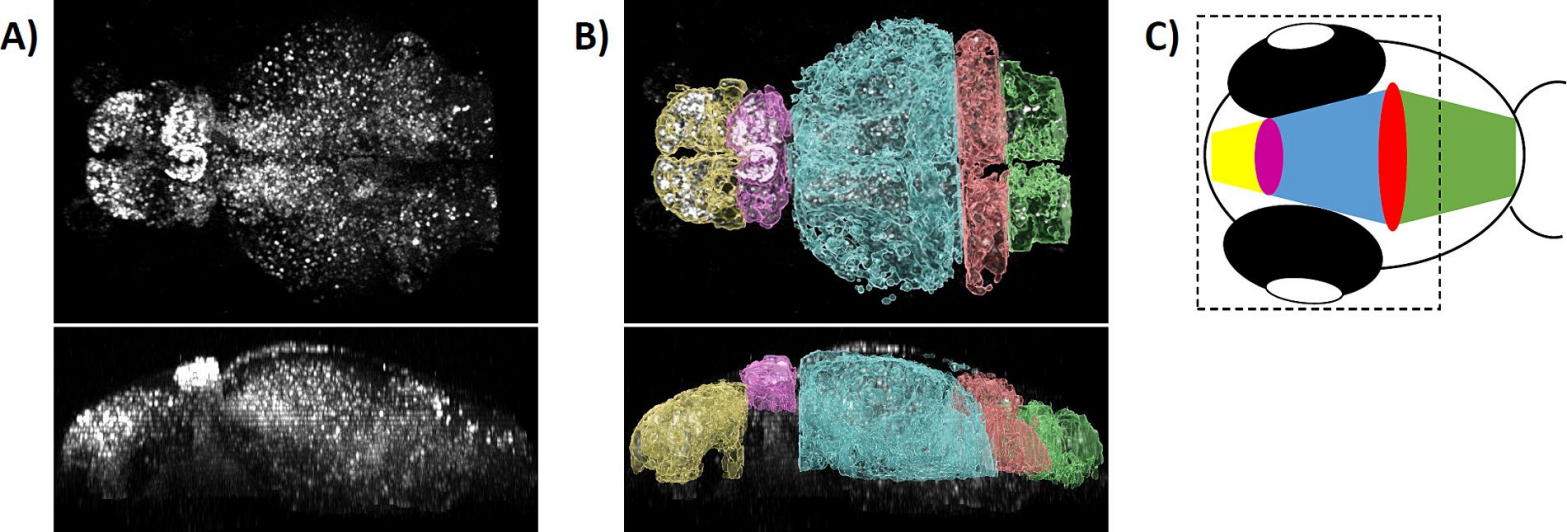
Modeling zebrafish brain of 4 dpf larvae. **(A)** A 3D stack of 4 dpf GCaMP fluorescent larval brain was generated and examined in dorsal (upper part) and lateral (lower part) view. **(B)** Based on anatomical features, five brain areas were designated roughly corresponding to pallium (yellow), habenula (violet), tectum (blue), cerebellum (red), and medulla (green), as shown as overlay with the raw data. **(C)** Schematically shown are the relative positions of designated brain areas in the head of the larva. The dashed line indicates the field of view in the microscope omitting the caudal part of the medulla.

**Figure 2:**
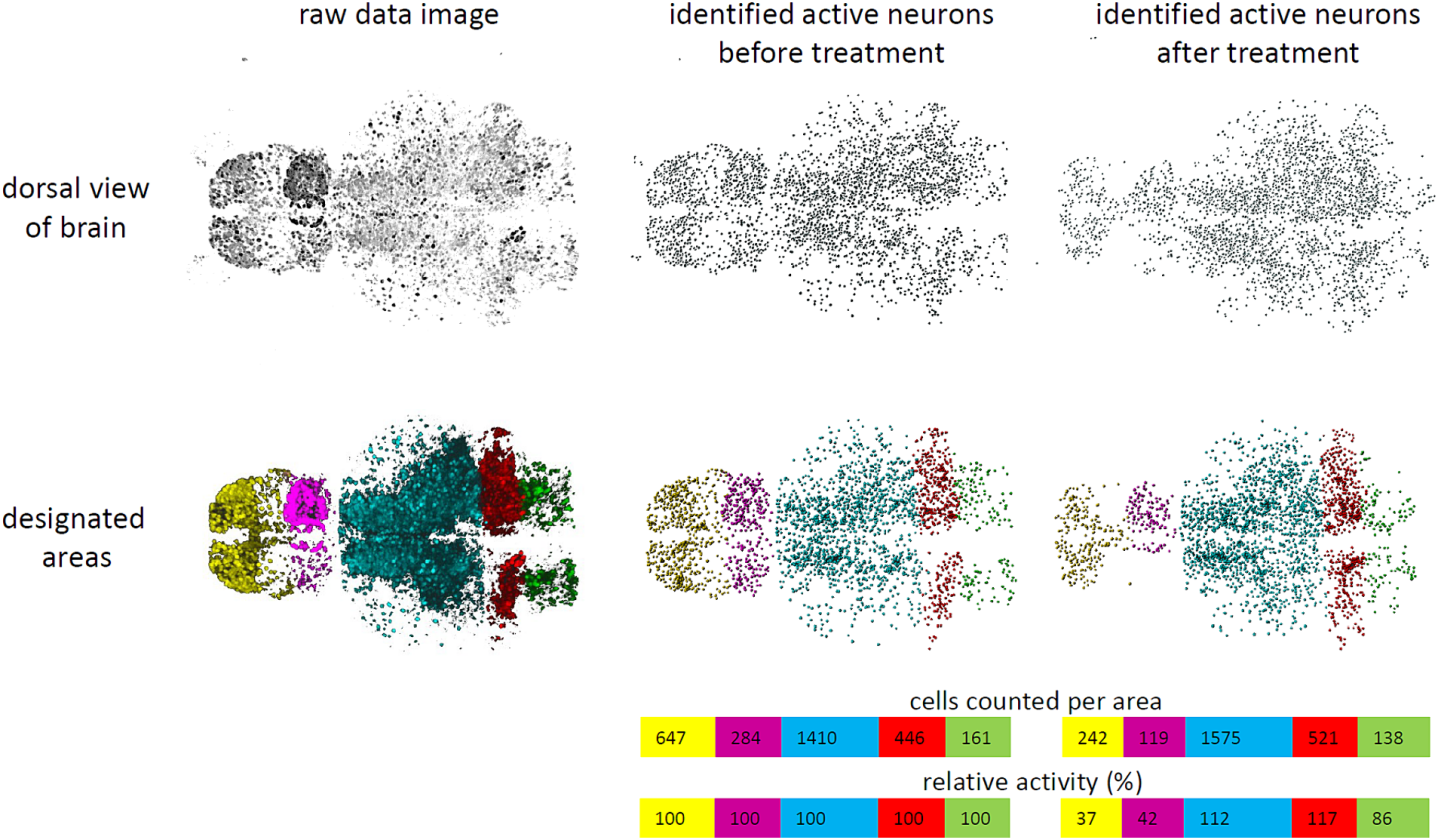
Schematic work flow to quantify neuronal activity. A work flow was established to quantify neuronal activity in larval zebrafish brains. Raw data images (upper left) were processed with Imaris software. Nuclei of individual neurons were identified and counted by applying the spotting function of Imaris software by a fluorescence threshold >7,000 and an expected diameter of 4 μm and represented as spots (upper middle). These were classified as “active” neurons. In addition, brain areas were designated as pallium (yellow), habenula (violet), tectum (blue), cerebellum (red), and medulla (green) (lower left). Therefore, active neurons could be counted per area (lower middle). Tracking cell numbers spotted over time allowed to determine changes in activity, when fluorescence of individual cells surpassed or failed the threshold (right). Relative activity was calculated based on the mean number of spots detected during baseline activity (colored bars).

GCaMP fluorophores were imported to the nucleus in all neurons due to the nuclear localization signal of the H2B-tag. This allowed for quantification of the overall neuronal activity by counting GCaMP-positive cells with high fluorescence intensity (Figures 1, 2). The volumes of different brain areas were determined according to anatomical characteristics based on their fluorescence, roughly corresponding to pallium (yellow), habenula (violet), tectum (blue), cerebellum (red), and medulla (green) (Figure 1) (Ronneberger et al., 2012). Settings for quantification were decided after initial pilot studies and before experiments were performed in the way to distinguish active and inactive neurons. For this, a protocol for automated quantification was set up with the Imaris software (Oxford Instruments). By applying the spot function to whole brain images with settings for expected spot size diameter of 4 μm and a threshold for fluorescence intensity mean > 7,000, neurons were classified as “active” and counted as spots (Figure 2; Supplementary Figures 2, 3). Fluorescent signals below the threshold were dismissed as “inactive” neurons or non-neuronal cells, even though a small population of active but low-expressing GCaMP neurons might be wrongly classified in that way. Based on pilot experiments, counting active cells based on the spot function was superior to quantifying neuronal activity based on fluorescence intensity mean alone (Supplementary Figure 4). This was due to residual background fluorescence in some tissues that obscured some smaller effects. Timelapse recordings with an average of less than 1,000 detected spots for baseline activity were considered of insufficient quality and were excluded from further evaluation. Then the number of active neurons were calculated for either the whole brain or a certain area and changes were tracked over time (Figure 2). As absolute numbers of detected spots varied greatly between individual larvae, relative neuronal activity was calculated by normalizing spot numbers to the average of 5-min baseline activity before treatment to allow for comparison between larvae.

### 2.6 Statistical methods

To determine if tricaine affected larvae behavior differently depending on age, the distribution of time needed to reach anesthesia was compared using a Log-rank test with Prism Software 9.3.1 (GraphPad). A 95% confidence interval was calculated by the method of Machin using the same software (Machin et al., 2006).

The main objective of the evaluation of the imaging of neuronal activity was to determine whether the application of tricaine creates a lasting and relevant decline of that activity over time. If this is the case, the neuronal activity would assume an asymptotic level after some time. However, it was unknown how long after its application tricaine is beginning to take effect.

Therefore, we made for statistical analysis the following assumptions: the development of the brain activity was divided into two parts. The first part consisted of a linear trend where no treatment effect is detectable. In the second part, the brain activity declined toward an asymptote. A change point divided the two parts. Since the exact shape of the brain activity development was unknown, we used two nonlinear models:

$$y\left(\mathrm{TIME}\right)=\mathrm{Asym}+\left(R0-\mathrm{Asym}\right)\;\exp.\left(-\exp\left(lrc\right)\;\mathrm{TIME}\right)+\varepsilon$$

In this asymptotic model, the parameter Asym stand for the value of y TIME- > ∞, i.e., the asymptotic value. The parameter R0 indicates the value of the activity at TIME = 0. The parameter lrc stands for the logarithm of the rate constant, which determines the velocity with which the neuronal activity reaches its asymptotic value. The other model was

$$y\left(\mathrm{TIME}\right)=a_{1}\mathrm{TIME}/\left(a_{2}+\mathrm{TIME}\right)+a_{3}+\varepsilon$$

Here, a_3_ represents the value of the activity at TIME = 0. The parameter a_1_ represents the asymptotic value minus a_3_, i.e., the difference between starting value and asymptotic value. The parameter a_2_ indicates the time at which the activity y(TIME) has lost half of the amount of the difference between starting value and asymptotic value.

The model parameters were estimated by the nonlinear least squares R function *nls* (R Core Team, 2022). For each potential change point and model, the residual sum of squares (RSS) was calculated for the composed model (linear + nonlinear part). The change point and model with the minimum RSS was selected to predict the mean population brain activity. To compare the resulting change in brain activity to that of the control individuals the linear trend of the latter was calculated.

The difference at the end of the period of measurement between the mean population brain activity of the control group (estimated by the linear model) and that of the treatment group (estimated by the nonlinear model), was thereby given. This difference was denoted as mean decline in brain activity (ΔBA).

To check whether the estimated mean decline of brain activity at the end of the measurement period could also be randomly obtained, a permutation test was applied. In brief, the individual measurements regarding the brain activity of control and tricaine treatments were randomly redistributed to control or treatment group and then calculated by which probability the same or a greater effect on brain activity could be achieved.

For this, the original data were composed of time series of brain activity of 20 control individuals and 13 individuals treated with tricaine. The null hypothesis was that the times series were from the same random distribution. Numbers from 1 to 33 were assigned to the individuals and by random permutation of these, they were randomly divided into two parts. The first part consisting of the first 20 numbers was regarded as control data, the second part as treatment data.

To this data set, the above described procedure to calculate a trendline and changepoint was again applied to determine a mean decline in brain activity. The whole procedure was repeated 10,000 times to derive a random distribution of ΔBA corresponding to the null hypothesis (ΔBA_perm_).

The number of events where by randomly redistributing ΔBA_perm_ was greater or equal than that estimated from the original data was counted (cases, where the model parameters could not be estimated, were not counted as greater or equal). This number divided by 10,000 was then regarded as *p* value.

An additional objective was to find out if the application of tricaine has any effect on the neuronal activity in different brain regions. To test the corresponding hypothesis, we applied a repeated measures ANOVA. For this purpose, we applied the R function *aov_car* from the package *afex*. From each activity value of the tricaine treatment at each time point, the mean value at the same time point of the control activities are substracted for this purpose. To find time points with potential differences between control and tricaine treatment in neuronal activities, we applied permutation tests for each time point. For this purpose, the R function *perm.test* of the package *exactRankTests* was applied.

## 3 Results

**Figure 3:**
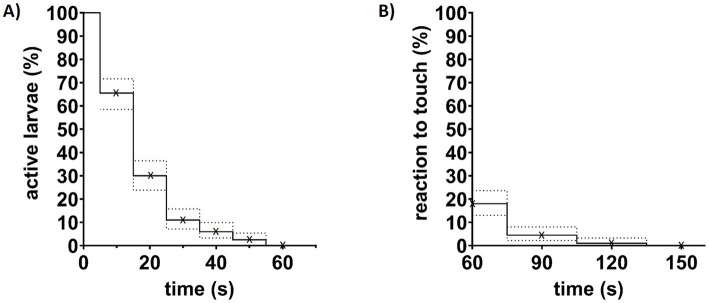
Effects of tricaine treatment on larvae behavior. **(A)** 200 larvae aged 4 or 5 dpf were observed for spontaneous body activity to control for effective anesthesia induction by tricaine. After treatment with 168 mg/L tricaine, they were observed for 60 s and the last timepoint of spontaneous activity was noted rounded up to the next 10 s. Shown are six groups of every 10 s steps indicated by (x). **(B)** 60, 90, 120, and 150 s after addition of tricaine, larvae were gently touched with a blunt metal poker (indicated by x). At 60 s, already 82% of larvae were unresponsive to touch, while the last 1% of larvae needed more than 120 s of treatment to reach that level of anesthesia. Dotted lines indicate interval of confidence (95%).

Tricaine activity can vary greatly depending on preparation and storage conditions. It loses activity quickly at room temperature and light (Harper, 2016). To ensure effective and comparable tricaine doses 100 4 dpf and 100 5 dpf wildtype larvae were individually immersed in 168 mg/L tricaine and were observed for 60 s for spontaneous activity like movements, tail flicks, or twitches. For 89% of larvae activity ceased within the first 30 s (Figure 3A). Only 2.5% still showed continued spontaneous activity for up to 60 s, 18% of them still reacted to touch at that time point (Figure 3). Within another 60 s (a total of 120 s after addition of tricaine), the number of larvae reacting to touch dropped to 1% (Figure 3B). After a total of 150 s of tricaine treatment, all larvae had lost balance and showed no response to touch (Figure 3B). No difference between 4 and 5 dpf larvae was observed in a Log-rank test both for spontaneous activity (*p* = 0.3582) or reaction elicited by touch (*p* = 0.5413) (Supplementary Figure 5).

**Figure 4:**
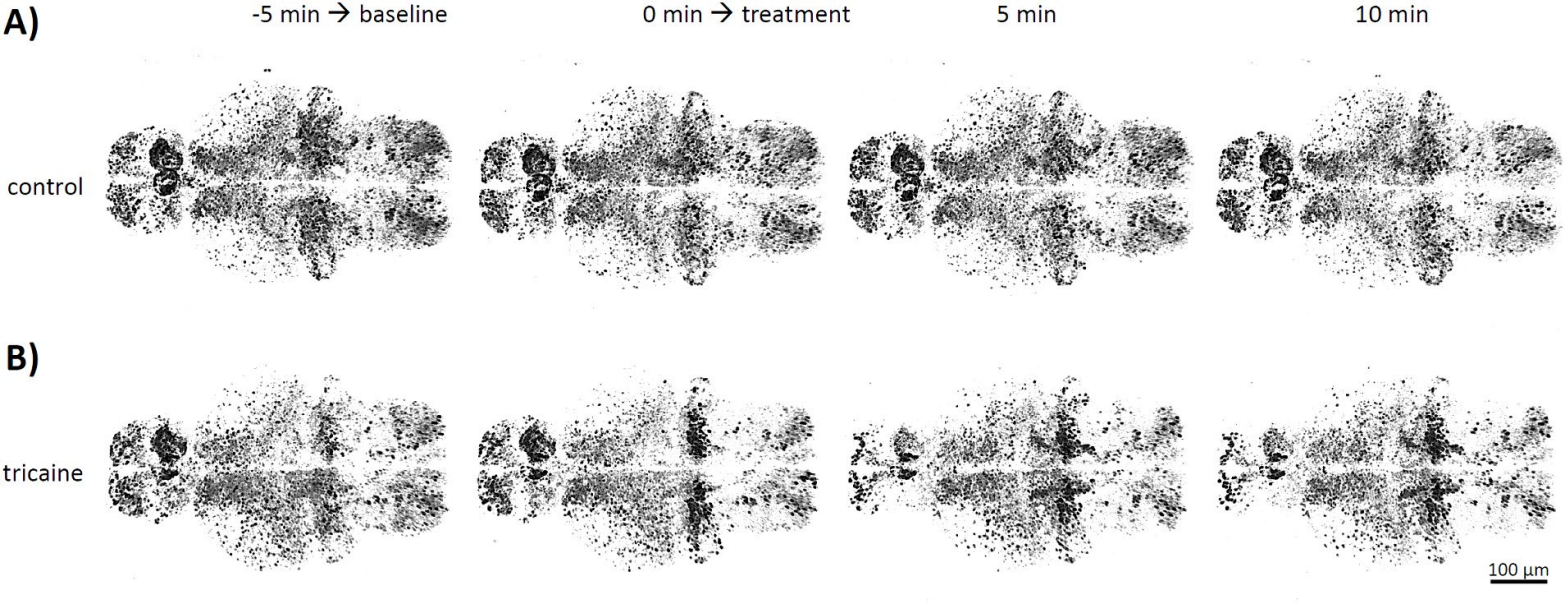
Timelapse imaging of neuronal activity after tricaine treatment. Tg(*elavl3*:H2B-GCaMP6s) larvae were imaged in confocal microscopy at 4 dpf. Stacks of whole larval brains were taken every minute with either E3 medium control (**A**, upper row) or 168 mg/L tricaine (**B**, lower row) as treatment. Shown are representative images of 3D stacks from a larval brain in dorsal view taken at the beginning and the end of the untreated phase used to determine baseline activity (−5 and 0 min), as well as images 5 and 10 min after treatment was applied.

Effects of tricaine on neuronal activity were investigated in the transgenic line Tg(*elavl3*:H2B-GCaMP6s) with the panneuronal expression of the fluorescent, intracellular calcium sensor GCaMP. Data of 5 min of measurement without treatment were used to calculate average baseline activity for each larva. Treatment in form of either E3 medium (Figure 4A, control) or 168 mg/L tricaine (Figure 4B, tricaine) was then given and time-lapse imaging continued for another 20 min to assess the lowest point of brain activity.

Continued imaging and sham treatment with E3 medium showed no obvious effect on overall brain activity or activity patterns (Figure 4A). In contrast, 5 min after tricaine treatment, a strong reduction of spontaneous activity in forebrain regions of pallium and habenula was observed (Figure 4B, 5 min). Effects on hindbrain cerebellum and medulla were less pronounced and with a higher variability between larvae.

**Figure 5:**
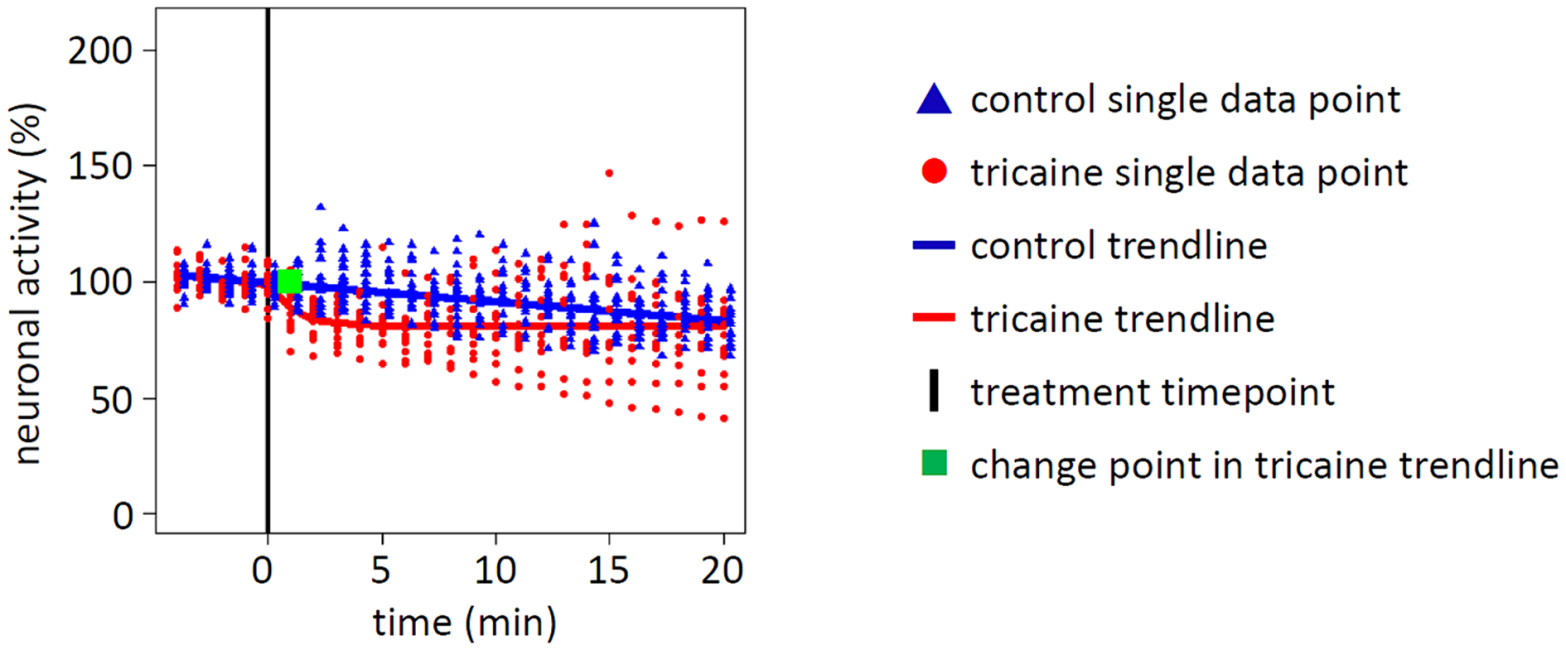
Effect of tricaine on whole brain neuronal activity. Neuronal activity of whole larval brains was determined by quantification of fluorescent nuclei. Values were normalized to average baseline activity of 5 min before treatment was applied (black line). Measurements of single larva are displayed for control (blue triangle, 20 individuals) or tricaine treatment (red dot, 13 individuals). From these measurements, a trendline for either control (blue line) or tricaine treatment (red line) was calculated. In addition, a change point was calculated for tricaine treatment (green square) for the time point when the treatment changed the trendline. Whereas the tricaine treatment initially induced a loss of neuronal activity, the trendline converged over time with the control treatment at 81% of baseline activity. Overall, there was no significant difference between both trendlines in the statistic model of the permutation test (*p* = 0.4237).

Quantification of spots considered as active neurons by Imaris software showed no overall significant change in tricaine treated larvae (*p* = 0.4237, Figure 5). Even though there was an initial loss of 20% of neuronal activity within 3 min after tricaine treatment, activity graphs of control and tricaine treatment aligned over time. The intermediate difference was confirmed in a *post-hoc* performed repeated measures ANOVA (*p* < 0.01) and the duration of this effect was 11 min as identified via permutation test (Supplementary Figure 6).

**Figure 6:**
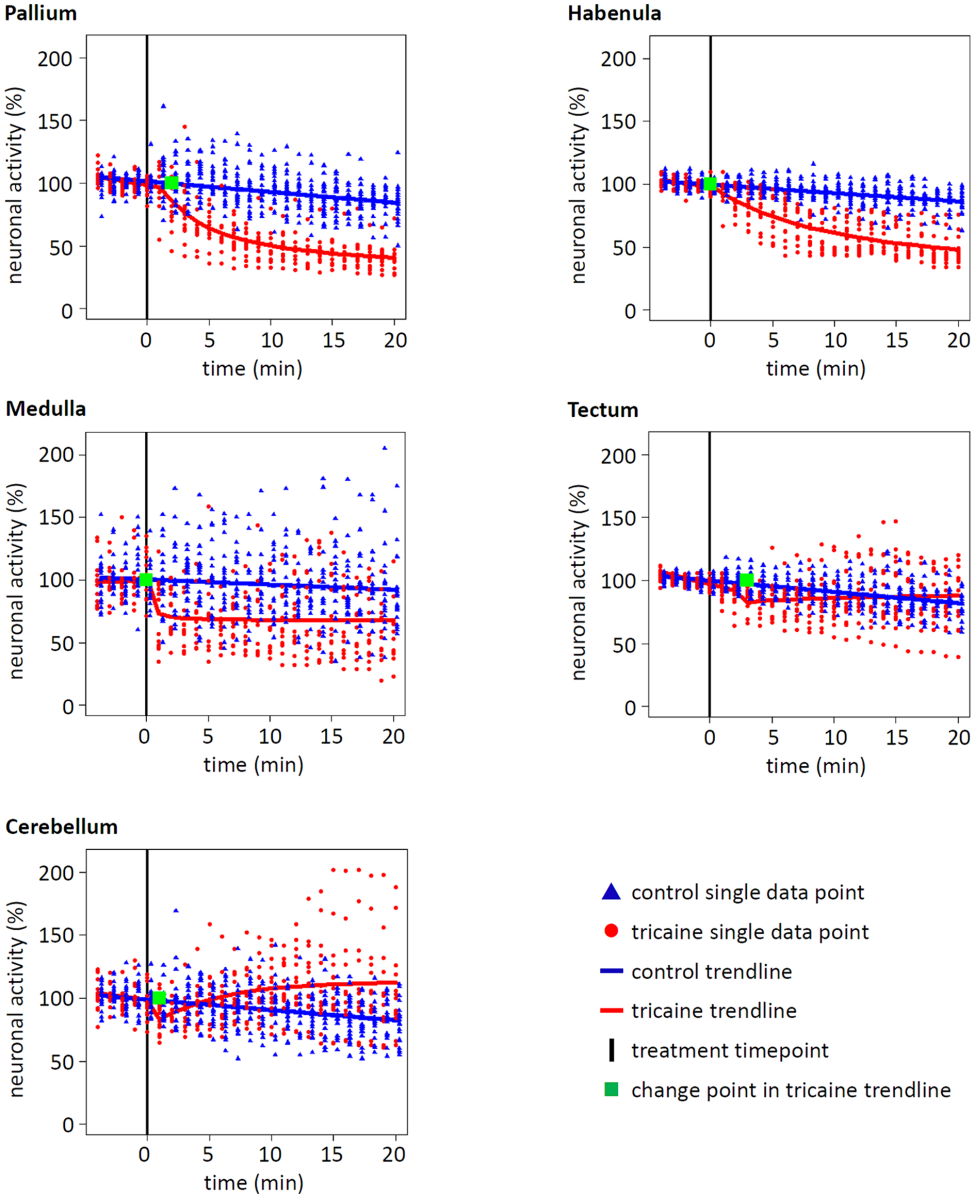
Effect of tricaine on neuronal activity of different brain areas. The neuronal activity of five brain areas was normalized to baseline and tracked for 25 min. After 5 min of baseline measurement, control or tricaine medium was added (black line). Each spot represents the relative activity of a single larva at the given time (control *n* = 20, tricaine *n* = 13). From these, a trendline and a change point were calculated (blue = control, red = tricaine, green = change point for tricaine treatment). For the brain regions pallium, habenula, and medulla, a significant decrease of activity after tricaine treatment in comparison to control treatment was calculated (see statistical methods, *p* values of <10^−4^, <10^−4^ and 0.0275, respectively). In contrast, activity in tectum and cerebellum was not significantly decreased over time (*p* value of 0.9765 and 0.7635, respectively). Whereas, in most brain regions, the tricaine treatment showed almost immediate effects, indicated by change points within 1 min after treatment, the change points of pallium and tectum manifested after 2 and 3 min of treatment, respectively.

Focusing on the activity of five designated brain areas, a different picture emerged compared to the whole brain activity (Figures 5, 6). For pallium and habenula, a strong decrease of activity after tricaine treatment was seen, with the trendline dropping to 41 and 48%, respectively, after 20 min of treatment compared to baseline activity (Figure 6). Medulla activity decreased as well but with 68% of neuronal activity after 20 min of treatment to a lesser extent compared to pallium or habenula. Even though these effects all started to manifest within the first 2 min after the addition of tricaine, as indicated by the calculated change point for the trend lines, the activity of the medulla plateaued immediately after the initial drop, while the activity of the pallium and the habenula kept decreasing for 10 and 20 min, respectively, before starting to plateau (Figure 6). In contrast, activity even seemed to increase in the cerebellum, plateauing at 112% of the baseline activity. Again, these findings were confirmed via *post-hoc* performed repeated measures ANOVA (*p* < 0.01) as well as the timing of the calculated change point was matched in a permutation test (Supplementary Figure 7).

Whereas all of the 20 larvae imaged for control were included in the final evaluation, five out of 18 measurements for tricaine treatment were disregarded after quantification analysis was completed. This was due to significant differences of effects seen with one specific charge of PharmaQ tricaine powder in the regions of tectum, cerebellum and medulla (Supplementary Figures 1A,B). As additional control, three more larvae were treated with chemical grade tricaine (Sigma Aldrich) for comparison to PharmaQ tricaine effects (Supplementary Figure 1C).

In addition, after initial evaluation was completed, another analysis was performed to determine if bleaching was the reason of activity loss in control treated larvae. For this, a single cell from a randomly picked control data set was tracked over time and its mean fluorescence intensity was determined. A 20% reduction of activity within the cell during imaging was observed (Supplementary Figure 8).

## 4 Discussion

Proper anesthesia protocols are required to avoid distress in the induction phase until immobility is reached as well as in the recovery phase. Additionally, a proper analgesia is essential for many procedures (Ohnesorge et al., 2021). Tricaine (MS-222) belongs to a family of anesthetics which have been widely studied but not completely understood (Chatigny et al., 2017). Therefore, it was the aim of this study to investigate whether tricaine (MS-222) acts rapidly and lastingly on the central nervous system of zebrafish larvae to estimate its anesthetic properties.

Typically, the effectiveness of anesthetic treatments is assessed via behavioral observations (Martins et al., 2016). As effectiveness of tricaine could vary based on preparation and storage compared to published data, we first tested its effects on behavior (Carter et al., 2011; Katz et al., 2020). In adult zebrafish, concentrations of 75–200 mg/L tricaine were shown to induce deep narcosis and anesthesia within 2 min (Grush et al., 2004; Chambel et al., 2015). These stages are characterized by loss of balance and missing reactions to postural changes and tactile stimuli (Martins et al., 2016).

In line with already published data, we observed a loss of spontaneous activity in 89% of 4 and 5 dpf larvae within 30 s and an absence of reactions to touch in less than 60 s for 82% of larvae (Leyden et al., 2022). Interestingly, 1% of larvae needed more than 120 s to reach a state considered as deep stage anesthesia. It remains unclear if this was due to variations in responsiveness to the drug or individual stress levels, but should be taken into account for designing potentially aversive or painful experiments.

Although behavioral models are well accepted to determine various stages of anesthesia, by the sole observation of the animal, paralyzing effects of a substance by acting on the periphery or muscles alone cannot be ruled out. It would also be possible that anesthetic effects of a given substance manifest later than its paralyzing effects (Machnik et al., 2023). To address these concerns, we used a neuronal-specific transgenic reporter line that reflects neuronal activity via changes in calcium concentrations.

It was concluded from the behavioral observations that with our lab protocols for preparation, storage, thawing and diluting of PharmaQ tricaine stocks a strong effect on neuronal activity was expected within 3 min after treatment. Since it was unclear which brain areas are relevant for tricaine treatment, an unbiased approach was chosen in which the entire brain was continuously imaged. Tricaine is known to act as a sodium channel blocker by prohibiting sodium entry into the nerve cell. It severely limits neuronal excitability and induces muscle relaxation (Frazier and Narahashi, 1975; Matthews and Varga, 2012). Therefore, we expected that tricaine treatment would rapidly induce a brain wide loss of neuronal activity. Instead, quantification showed only sparse brain wide effects. Even though there was initially a 20% drop of the neuronal activity, the activity levels of control and tricaine treated larvae converged over time. A more nuanced picture emerged at closer inspection. Tricaine treatment significantly changed neuronal activity patterns in specific brain regions. While smaller regions like pallium, habenula and medulla quickly showed a reduced activity, for large parts of the brain encompassing tectum and cerebellum the initial hypothesis that these regions would decrease in activity had to be rejected.

The reduced neuronal activity in regions of the forebrain is a good indicator for tricaine’s anesthetic properties. Even though it is not known yet which structures in teleosts have functions homologous to the mammalian neocortex, it was shown that most cognitive functions, like perception of time, depend on forebrain activity (Cheng et al., 2014; Salas et al., 2003). Therefore, it is more likely that zebrafish actually experience unconsciousness if the forebrain activity is reduced. In addition, our calculations showed that the trendline changed direction rapidly after tricaine treatment, indicated by change points within 2 min and thereby within the same timeframe as behavioral changes. Even though it is impossible to say which level of neuronal activity represents unconsciousness, at least tricaine showed immediate effects on the central nervous system, which is a second central requirement for good anesthetic properties.

In contrast, our method failed to show a lasting effect of tricaine treatment on tectum activity while a decrease of detectable cells in pretectum had been shown to occur after 7 min of tricaine exposure (Leyden et al., 2022). One reason for the discrepancy could be different stages of brain development, as we used 4 dpf larvae raised at 28°C while Leyden et al. used 5–7 dpf larvae raised at 29°C and it has been shown that during 5–6 dpf the tectum undergoes significant changes in spontaneous activity during reorganization of its functions of processing visual stimuli (Avitan et al., 2017). Furthermore, Leyden et al. investigated responses to visual stimuli, which could be blocked by tricaine in the retina and the effect would then be inherited in the optic tectum. They concluded from their analysis that even though behavioral responses ceased after tricaine treatment, that visual stimuli were still processed. Therefore, future experiments should include a more detailed analysis of neuronal activity, e.g., regarding changes of intensity and frequency of forebrain cells as reaction to sensory stimuli like olfaction to determine to which degree sensory input is still processed (Herrera et al., 2021).

The increase of neuronal activity in the cerebellum was quite unexpected and this finding was not in the scope of the initial working hypothesis. The beforehand chosen model could not verify the statistical significance. Still, a *post hoc* analysis of the data confirmed the increase of cerebellum activity. Interestingly this increase manifested much later than the effects in other brain regions. Although the change point appeared after just 1 min after tricaine addition, it took 6 min of treatment until the activity level raised above control level. It could be speculated that the reason behind the slow buildup of cerebellum activity is due to its function as sensorimotor processor (Lin et al., 2020). Here, the cerebellar activity could represent a feedback loop as increasingly deeper stages of anesthesia are accompanied with paralysis, decreased heartbeat and loss of muscle tone and thereby hampering cerebellar function of body homeostasis. An indication for this could be the aversive reaction of zebrafish to tricaine, resulting in hypertaxia and tachypnea before the onset of anesthesia (Matthews and Varga, 2012). The sensory input of tricaine as a stressor could remain even though the fish is unconscious. Still, additional research is needed to investigate the underlying mechanisms.

Nevertheless, further functional interconnections between other brain regions possibly involved in (un)consciousness have not been examined neither in the present study nor in other studies (Leyden et al., 2022; Loring et al., 2020). Identifying the brain regions involved in pain perception and anti-nociception thus seems to be an urgent need. While behavioral studies provide some insights (Ehrlich et al., 2019), the brain activity during anesthesia recovery, as well as during other anesthesia-related effects such as anxiolysis, amnesia, and immobility, still requires further investigation. This knowledge is crucial to refine anesthesia protocols for zebrafish.

Taking into account, the various known side effects of MS-222, e.g., aversion, epidermal and corneal lesions, hypoxemia, decreased heart rate and death, evaluating other anesthesia protocols with our proposed model is required to determine a safe anesthesia with possibly lower side effects (Huang et al., 2010; Wong et al., 2014; Readman et al., 2013; Davis et al., 2008). Good candidates for this might be lidocaine, propofol, or combinations thereof. They were shown to be suitable alternatives to tricaine with rapid induction of anesthesia and analgesic properties (Bernstein et al., 1997; Rombough, 2007; Collymore, 2020; Collymore et al., 2014; Ferreira et al., 2022; Martins et al., 2018; Martins et al., 2016).

A limitation of the present study is that our results only relate to anesthesia in larvae. Further research is needed to elucidate the anesthetic effects of tricaine or its alternatives in the adolescent and adult zebrafish brain.

In summary, we investigated whole larval brain activity in an unbiased approach via calcium imaging of neuronal cells and could show that tricaine affects the central nervous system within 1 min in accordance with behavioral observations. Surprisingly different effects on neuronal activity were observed depending on the specific brain region. While in pallium, habenula and medulla activity quickly decreased, tectum remained mostly unaffected and cerebellum showed a potential increase of activity over a longer period. In conclusion, tricaine can be considered a safe anesthetic that on average acts fast on the central nervous system of zebrafish larvae and not only as a paralytic in the periphery. Still, to ensure sufficient reduction of neuronal activity in the central nervous system, higher doses than 168 mg/L or incubation time of at least 2 min should be considered and compared with alternatives like lidocaine and propofol in future experiments for an increased safety regarding animal welfare.

## Data Availability

The raw data supporting the conclusions of this article will be made available by the authors, without undue reservation.
