# Supplementary material for: Assessment of the effect of tricaine (MS-222)-induced anesthesia on brain-wide neuronal activity of zebrafish (Danio rerio) larvae
Source: Front Neurosci. 2024 Sep 24;18:1456322. doi: 10.3389/fnins.2024.1456322 (PMC11458748; doi:10.3389/fnins.2024.1456322)
Supplement: Supplementary file 2 [file Data_Sheet_1.docx]

Supplementary Material

# Supplementary Figures and Tables

## Supplementary Figure 1


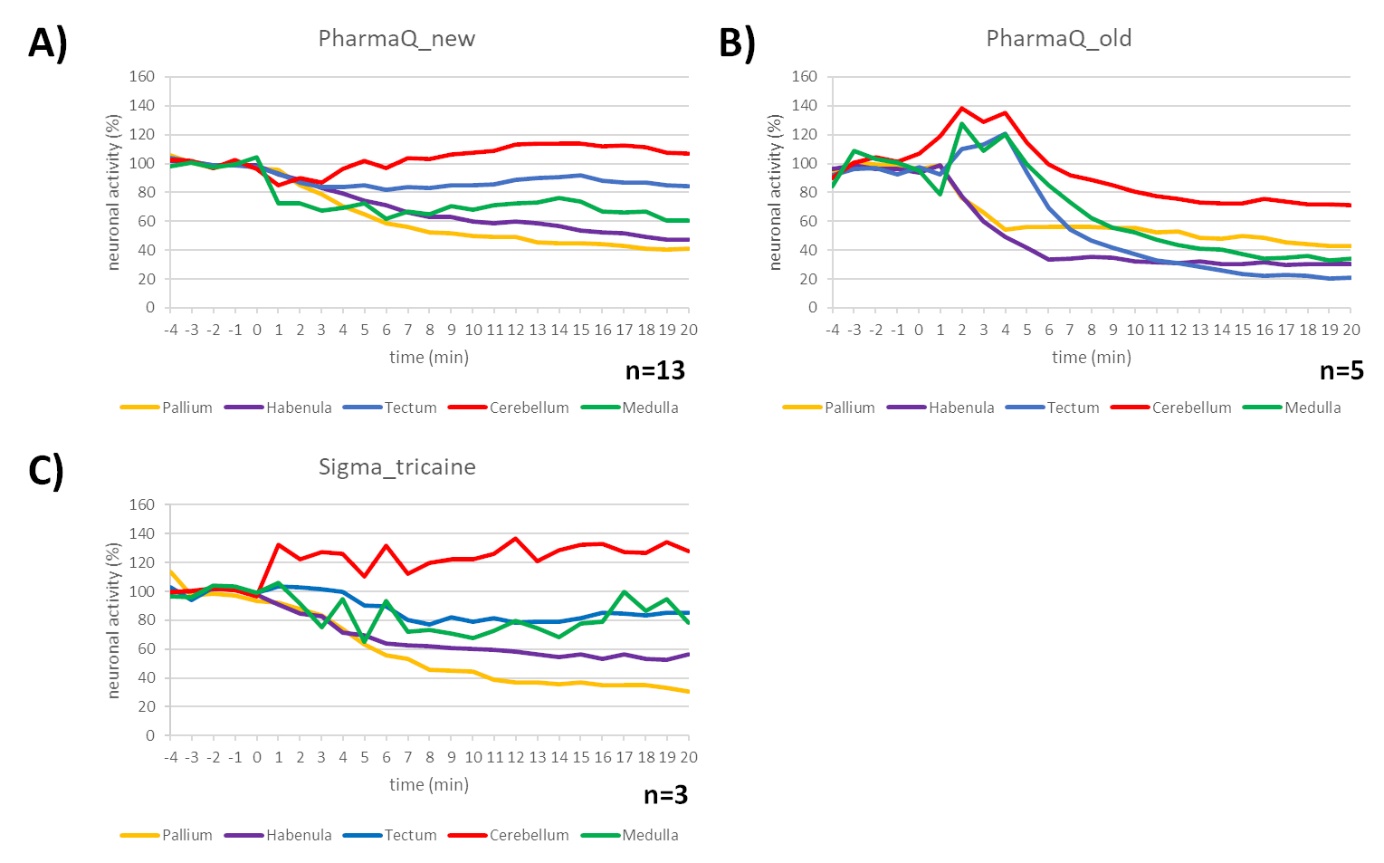


**Sup. 1: Different patterns of activity in older charge of PharmaQ tricaine**. Some activity patterns were observed only in pilot studies and the first experiments, shown here as mean values of the raw data. In A) is the data shown used throughout the rest of the paper with 168 mg/L tricaine treatment (n=13). B) In contrast to these, first studies showed an initial increase of neuronal activity in tectum, cerebellum and medulla in the first 5 minutes after adding tricaine only to be more reduced in activity at later timepoints (n=5). After starting to use a new charge of fresh PharmaQ tricaine powder, these observations could not be reproduced and despite thorough control testing, no other reason for the change of activity patterns could be determined. In addition, no substance of the first tricaine powder charge was left to compare in more detail reactions to different charges of tricaine treatment. C) As an additional control, tricaine of chemical grade (Sigma-Aldrich) was tested (n=3), with activity patterns more similar to the newer PharmaQ charge. From this it was concluded, that these results were more reliable to base conclusions on and the data from pilot studies B) and C) was not included in the final assessment.

## Supplementary Figure 2


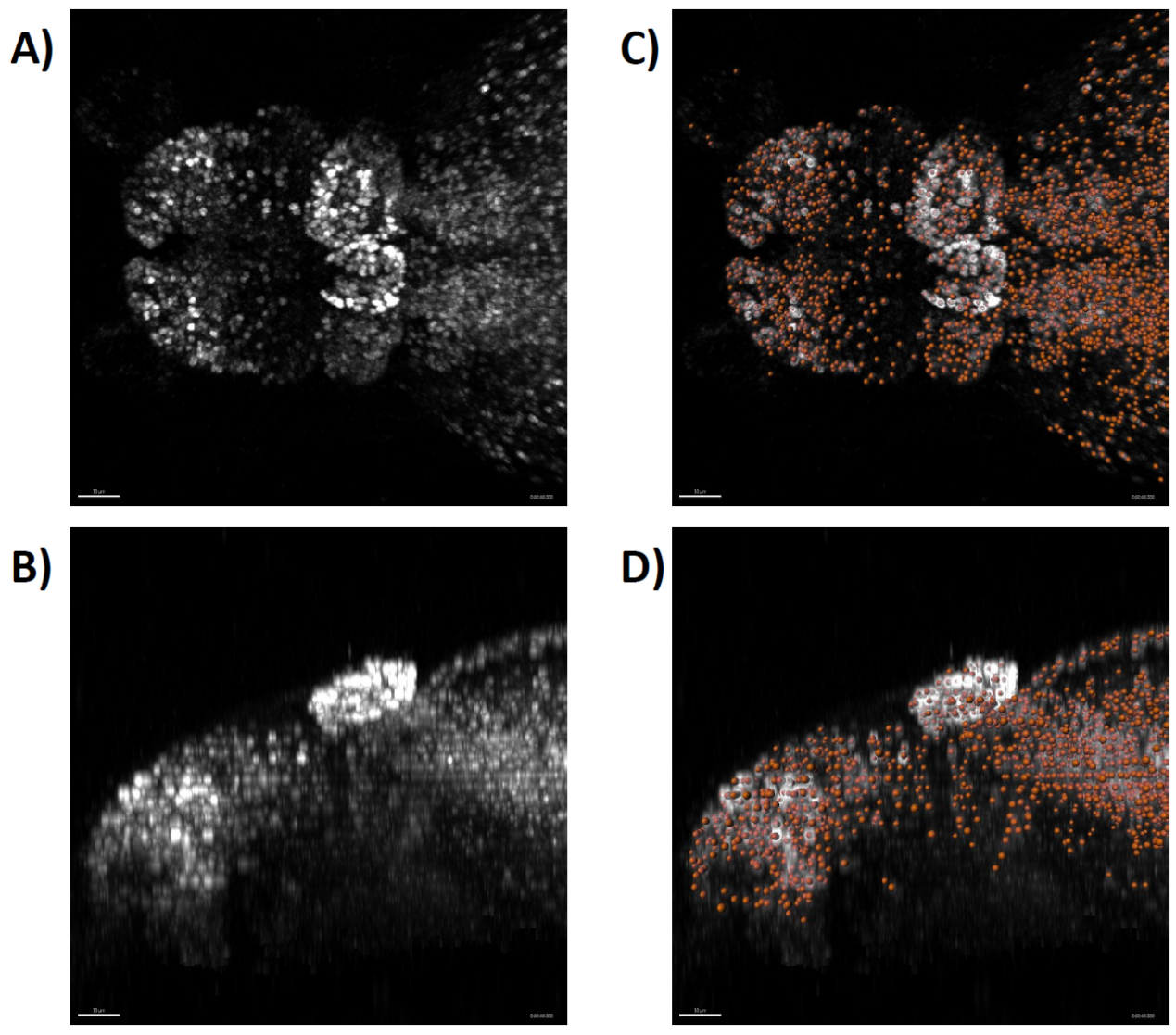


**Sup. 2: Identification and count of neurons**. For each imaged larva spot function of Imaris software was used with settings for expected spot size diameter of 4 µm and fluorescence intensity mean > 7000, to detect and count number of neurons. Images were taken as snapshots at 0 min from the movie in the supplements, before tricaine was added. Shown are parts of the forebrain in A) dorsal and B) lateral view with spots matching the neuronal nuclei (C+D). Scale bar equals 30 µm.

## Supplementary Figure 3


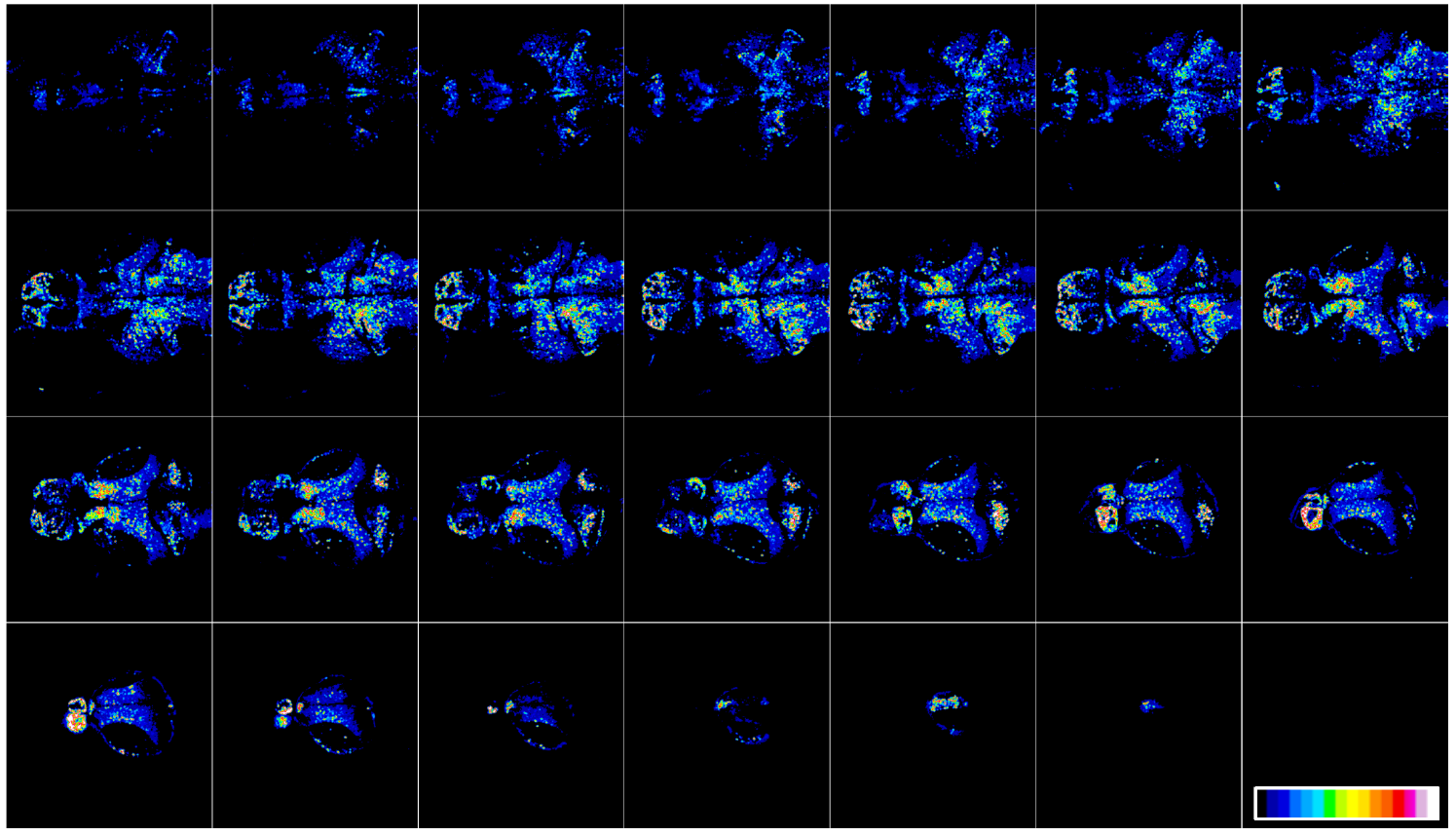


**Sup. 3: Identification of affected brain regions**. A standard deviation projection of a tricaine treated larvae was created using ImageJ v1.53. Hereby the change in fluorescence intensity over the timecourse was visualized, allowing to identify brain regions with the greatest changes. The montage shows the individual imaging slices from most ventral (upper left) to dorsal (lower right) position. The scale bar indicates magnitude of differences in between the individual timepoints, with white being the highest.

## Supplementary Figure 4


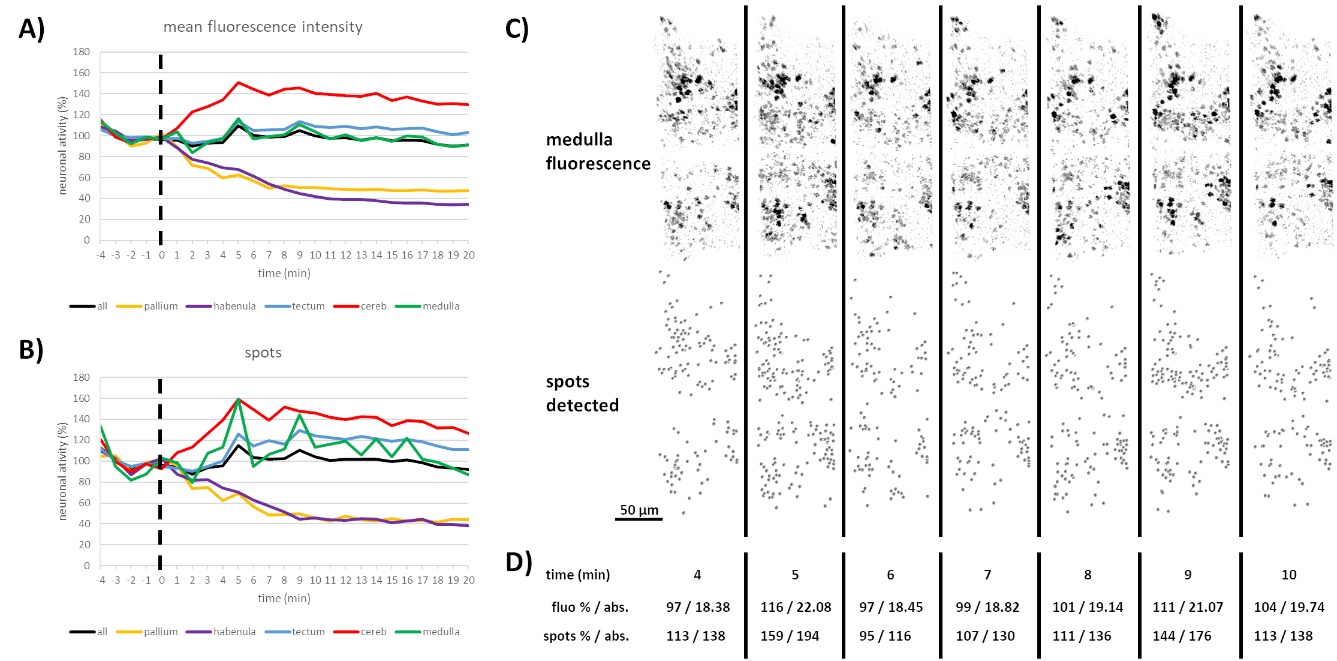


**Sup. 4: Quantification via mean fluorescence intensity vs. spot function.** The optimal method for quantification was determined in pilot experiments and it was concluded that the spot function is preferable over mean fluorescence intensity. As an example, the quantifications of neuronal activity of a randomly picked single tricaine-treated larva from the dataset are shown, measured either by A) mean fluorescence intensity or B) spots counted. In both cases data was normalized against baseline activity. Overall, both methods gave very similar results by reliably mirroring the major trends of activity. In this individual case, some differences appeared for quantification of medulla activity (green), with small peaks of activity detected by the spot function at 5 and 9 minutes after tricaine treatment. C) Visual inspection of the fluorescent images for the medulla region at these timepoints confirmed that indeed a small increase of activity took place (upper panels), noticeable by more black and dark grey nuclei. D) Due to a better signal to noise ratio these peaks of activity were more truthfully shown by the spot function and less so by measuring mean fluorescence intensity, as quantified by relative (or absolute) values of fluorescence and counted spots at the given timepoints (4 – 10 minutes).

## Supplementary Figure 5

**Sup. 5: Effect of tricaine on behavior of 4 or 5 dpf larvae.** The effect of 168 mg/L tricaine was determined on 100 of each 4 or 5 dpf larvae. No significant difference was seen in a Log-rank test for either A) spontaneous activity (p = 0.3582) or B) reaction to touch (p = 0.5413) between these two developmental stages.

## Supplementary Figure 6

**
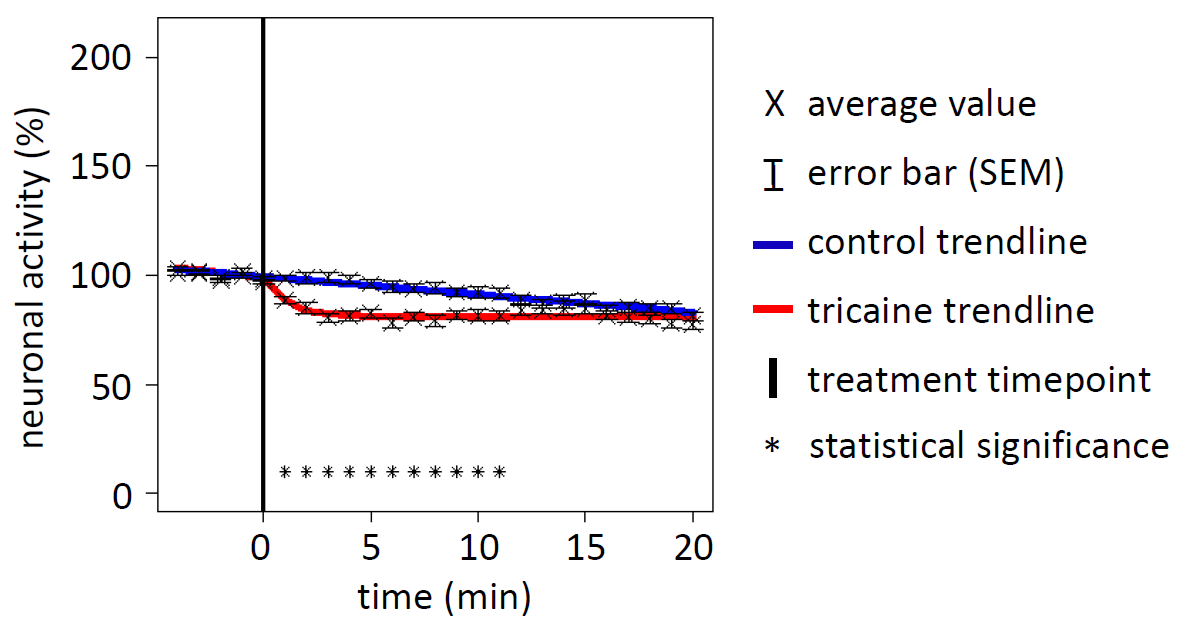
**

**Sup. 6: Effect of tricaine on whole brain neuronal activity.** To confirm quality of the chosen statistical model, the calculated trendlines for control (blue) or tricaine treatment (red) were tested post hoc in additional statistical tests. A repeated measures ANOVA confirmed a difference between the two data sets (p < 0.01). Both trendlines run close to average values (x) and the standard error of the mean (SEM). Post hoc analysis via permutation test showed a difference during the first 11 minutes after treatment (*).

## Supplementary Figure 7

**Sup. 7: Effect of tricaine on neuronal activity of different brain areas.** To confirm quality of the chosen statistical model, the calculated trendlines for control (blue) or tricaine treatment (red) were tested post hoc in additional statistical tests. A repeated measures ANOVA confirmed a difference between the two data sets in all brain regions (p < 0.01). The trendlines run close to average values (x) and the standard error of the mean (SEM). Post hoc analysis via permutation test showed a difference in tectum activity during the first 8 minutes after treatment (*).

## Supplementary Figure 8


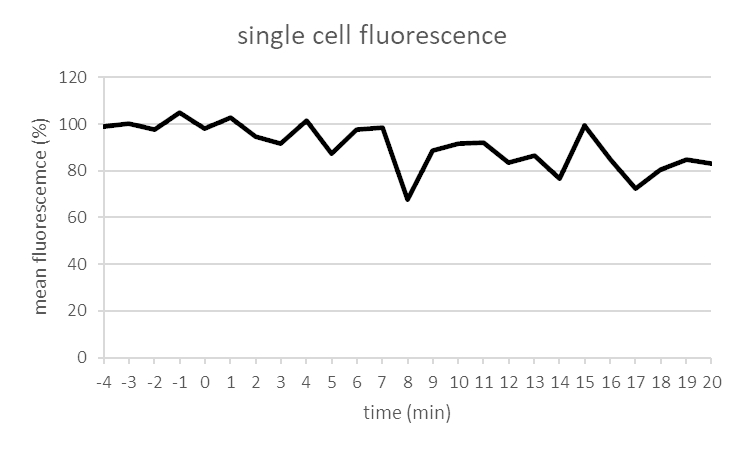


**Sup. 8: Bleaching effect in single cell.** It was a trend observed in control measurements that the neuronal activity drops over time to 80%. It was unclear if this was due to bleaching or other effects like continuous habituation of the larvae to the experimental conditions. To investigate this, a single cell was tracked in a randomly picked larva of the control group and the mean fluorescence was measured over time. Here, the same effect was observed as for the whole brain activity. Therefore, it was concluded, that the loss was rather due to bleaching than reduction of neuronal activity.
